# Administration of ketogenic intervention as a potential treatment during post-traumatic brain injury recovery: a scoping review

**DOI:** 10.3389/fnut.2026.1848682

**Published:** 2026-05-29

**Authors:** Daaniyal Y. Quddus, Serhat Aydin, Ayesha Akbar Waheed, Qazi Zeeshan, D. Kojo Hamilton, Nitin Agarwal, Joseph C. Maroon

**Affiliations:** 1 Department of Neurological Surgery, University of Pittsburgh School of Medicine, Pittsburgh, PA, United States; 2 Department of Neurological Surgery, University of Pittsburgh Medical Center, Pittsburgh, PA, United States; 3 Department of Neurological Surgery, Veterans Affairs Pittsburgh Healthcare System, Pittsburgh, PA, United States

**Keywords:** evidence mapping, exogenous ketones, ketogenic diet, ketogenic intervention, metabolic therapy, scoping review, therapeutic ketosis, traumatic brain injury

## Abstract

**Background:**

Traumatic brain injury (TBI) affects 69 million individuals annually and represents a major global health burden. Ketogenic interventions have emerged as a potential therapy to address deficits following TBI. This scoping review updates previous work to synthesize evidence on ketogenic therapies for TBI, examining mechanistic insights, clinical feasibility, and barriers.

**Methods:**

A systematic search was conducted following the methodological framework of Arksey and O’Malley (2005) and JBI guidance. Findings are reported in accordance with PRISMA 2020 guidelines complemented by the PRISMA Extension for Scoping Reviews (PRISMA-ScR). Studies were included if they used TBI models in human or animal subjects; investigated ketogenic interventions; reported quantitative clinical, functional, or metabolic outcomes; and enrolled more than five participants in clinical trials. Studies were excluded if they were conducted *in vitro*, did not involve TBI or ketogenic interventions, were not original research, were duplicate publications, or lacked English full text manuscripts.

**Results:**

Out of 421 studies screened, 32 studies met the inclusion criteria. 25 animal studies and 7 human studies were analyzed. 68.0% of animal studies used exclusively male specimens. The total pooled male-to-female ratio across human studies was 53:24, reflecting an approximately twofold predominance of male participants. Studies were evaluated using clusters of the most-assessed frameworks. Animal investigations evaluated metabolic pathways in 23 out of 25 (92.0%) studies, neuroprotective effects in 15 out of 25 (60.0%) studies, and behavioral assays in 13 out of 25 (52.0%) studies. Human studies assessed feasibility in 7 out of 7 (100%) studies, safety in 5 out of 7 (83.33%) studies, and efficacy in 4 out of 7 (66.67%) studies. Clinical trials consistently demonstrated feasibility in monitored settings.

**Conclusion:**

Ketogenic interventions improve metabolic dysfunction in preclinical models with variable neuroprotective and functional effects. Clinical evidence demonstrates feasibility but lacks adequately powered efficacy trials. Larger controlled trials with standardized endpoints with balanced age and sex representation are needed to determine efficacy.

## Introduction

Traumatic brain injury (TBI) is a major contributor to global disability, accounting for more than 8 million years of living with impairment annually (1). Approximately 69 million individuals experience TBI each year, many of whom develop long-term cognitive, behavioral, or physical impairments (2). Thus, it is highlighted as a public-health burden across the globe due to high treatment costs and disparity of protocols used in medical centers (2, 3).

**Figure 1:**
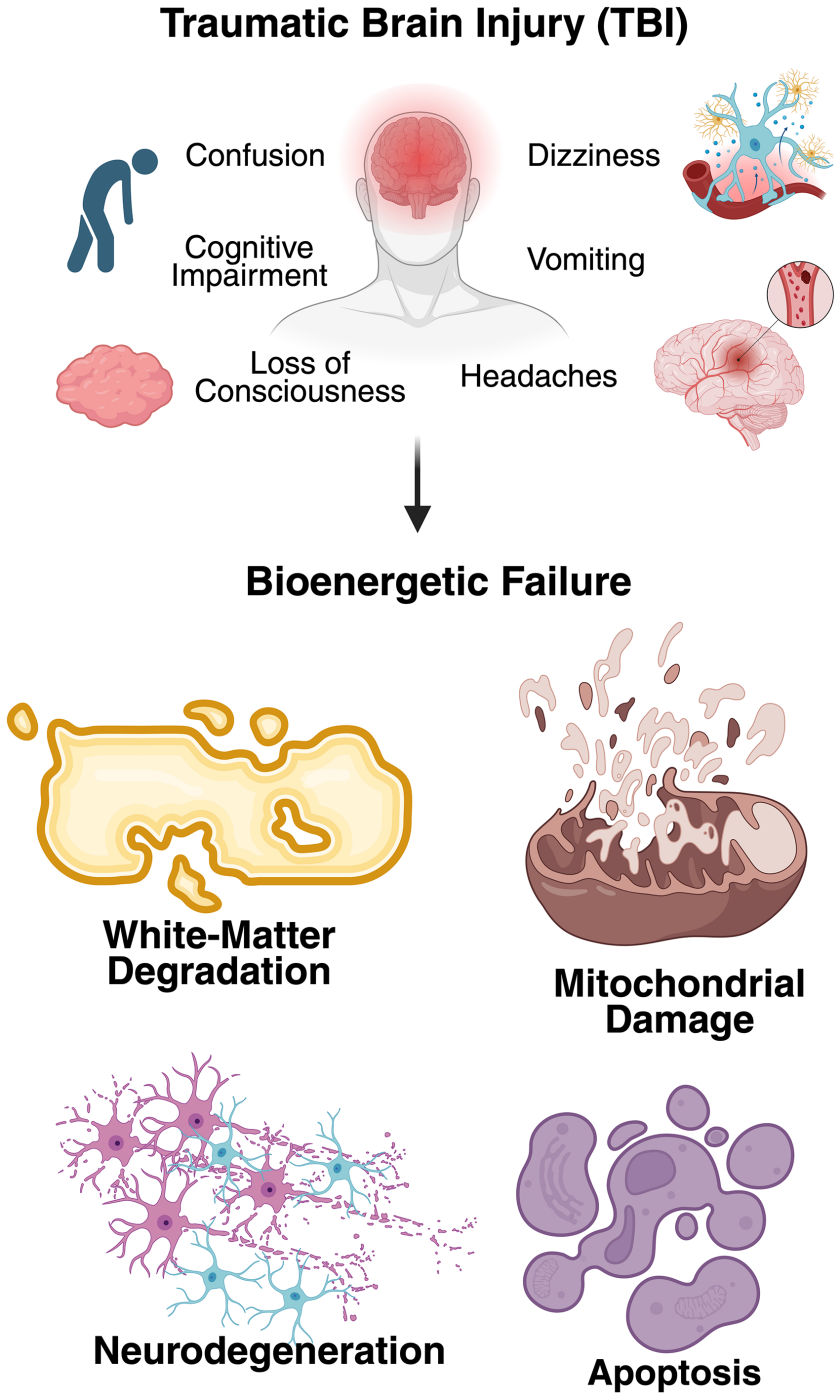
Biological consequences following traumatic brain injury (TBI). TBI presents acutely with symptoms including confusion, dizziness, cognitive impairment, headaches, vomiting, and loss of consciousness, which converge on a state of bioenergetic failure. This energetic collapse drives downstream pathological outcomes. (TBI; Traumatic Brain Injury). Created in BioRender. Quddus, D. (2026) https://BioRender.com/kr5wipr.

TBI triggers secondary injury mechanisms, including cerebral ischemia, glutamate-mediated excitotoxicity, oxidative stress, and neuroinflammation, all of which can exacerbate neuronal dysfunction beyond the initial insult (4, 5), (Figure 1). During the sub-acute and chronic phases of TBI, damaged neurons demonstrate dynamic structural and functional reorganization at anatomical, molecular, and functional levels. This reflects degeneration and compensatory adaptations across circuits affected by the injury. Axonal damage and white matter tract degeneration impair interregional connectivity, producing slowed information processing and executive dysfunction (6). Over time, persistent mitochondrial dysfunction further compromises ATP availability, accelerating vulnerability to neurodegenerative processes (7). Secondary injury cascades that develop minutes to days after the primary insult may affect long-term rehabilitation efforts, underscoring the necessity for clinical intervention. Therefore, without proper intervention, compensatory interventions might fail, resulting in lifelong debilitation and/or death (5, 8).

**Figure 2:**
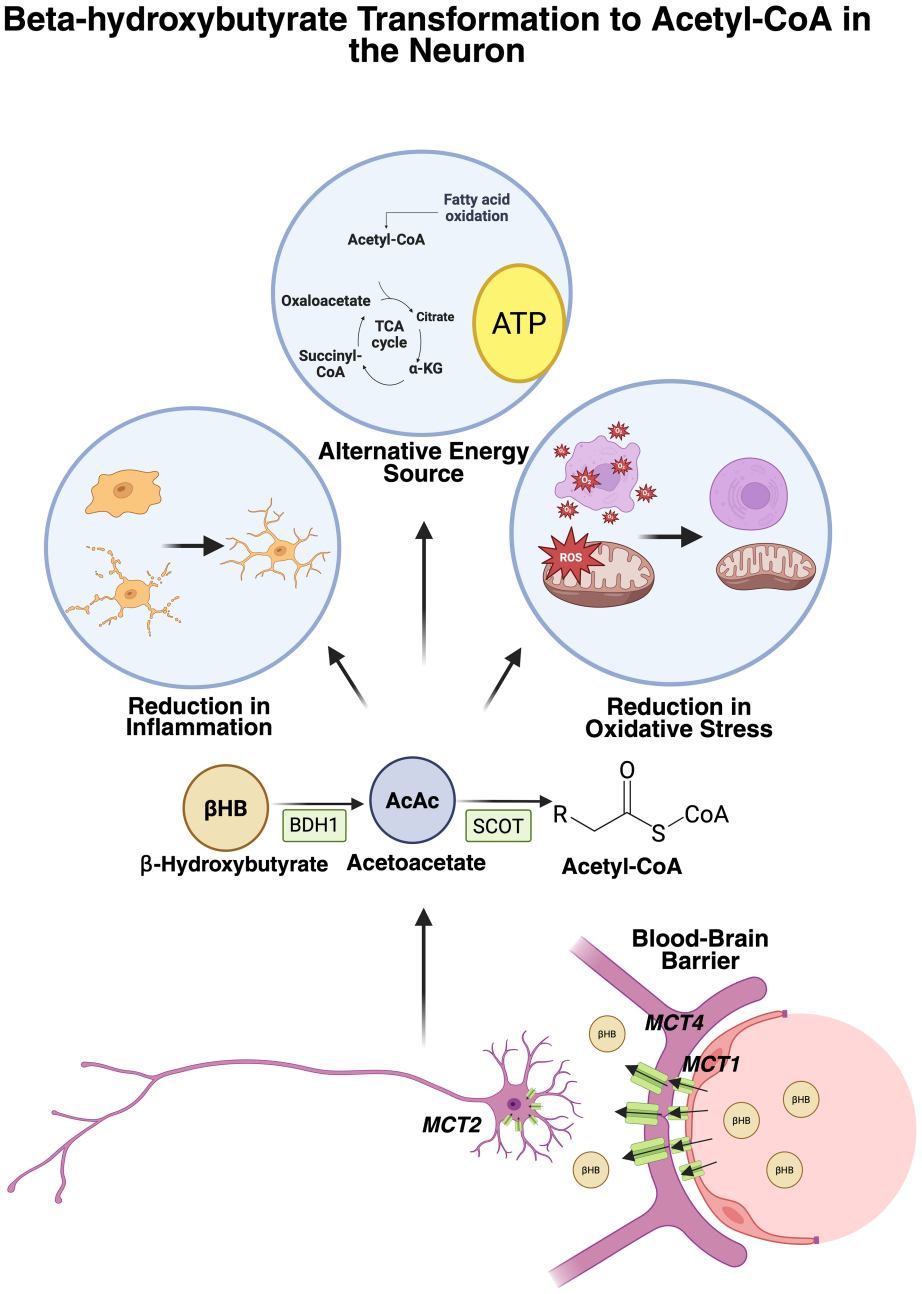
Mechanisms of beta-hydroxybutyrate transport following traumatic brain injury and ketogenic intervention. Circulating βHB crosses the blood–brain barrier via monocarboxylate transporters (MCT1/4 on the endothelium; MCT2 on neurons) and is converted sequentially to acetoacetate (AcAc) by BDH1 and then to acetyl-CoA by SCOT. Acetyl-CoA enters the TCA cycle to generate ATP via oxidative phosphorylation. (βHB; Beta-Hydroxybutyrate, BDH1; Beta-Hydroxybutyrate Dehydrogenase 1, AcAc; Acetoacetate, SCOT; Succinyl-CoA:3-Oxoacid CoA Transferase, MCT1; Monocarboxylate Transporter 1, MCT2; Monocarboxylate Transporter 2, MCT4; Monocarboxylate Transporter 4, TCA; Tricarboxylic Acid, *α*-KG; Alpha-Ketoglutarate, ATP; Adenosine Triphosphate, ROS; Reactive Oxygen Species). Created in BioRender. Quddus, D. (2026) https://BioRender.com/kr5wipr.

Cognitive rehabilitation therapy is utilized as a strategy for TBI patients to address long-term issues with memory, executive function, and problem-solving abilities (5, 9). In parallel, hyperbaric oxygen therapy (HBOT) has gained attention as a potential treatment for a range of neurological and psychological symptoms collectively described as post-concussion syndrome (10). As interest in adjunctive and alternative therapeutic strategies grows, metabolic interventions have become an area of particular focus. Since the publication of the last scoping review, research on ketogenic approaches to TBI has expanded substantially, driven by the growing recognition of the brain’s metabolic vulnerability following injury (11), (Figure 2).

Given this evolving evidence base, there is a need to synthesize emerging data and clarify how ketogenic interventions may complement established rehabilitation strategies. The present study aims to update and extend previous work by summarizing current mechanistic and clinical insights into ketogenic interventions, examining sources of heterogeneity across studies, and identifying the key barriers that must be addressed to facilitate their successful clinical translation. The primary objective of this scoping review was to map and synthesize existing evidence on ketogenic interventions in human and animal models of TBI. Secondary objectives were to characterize methodological heterogeneity across study designs, identify gaps in representation, and underline priorities for future clinical investigation.

## Methods

This study was reported in accordance with PRISMA 2020 as the foundational reporting guideline, complemented by the PRISMA Extension for Scoping Reviews (12, 13). The review process was based on the methodological framework proposed by Arksey and O’Malley (2005) and conducted in accordance with Joanna Briggs Institute guidance (14–16). The risk of bias assessment and PROSPERO registration were not conducted due to the nature of this study being a scoping review.

### Search strategy

A systematic literature search was performed on April 19, 2026, using medical subject headings (MeSH) related to the ketogenic diet and traumatic brain injuries. Studies were identified using PubMed and Web of Science databases. The search query used for PubMed was as follows: (“Diet, Ketogenic”[Mesh] OR ketogen*[tiab] OR “ketogenic diet”[tiab] OR ketosis[tiab] OR “Ketone Bodies”[Mesh] OR keton*[tiab]) AND (“Brain Injuries, Traumatic”[Mesh] OR “Craniocerebral Trauma” [Mesh] OR “Concussion” OR (traumatic[tiab] AND brain injur*[tiab]) OR head injur*[tiab] OR TBI[tiab] OR mTBI[tiab] OR concuss*[tiab] OR “diffuse axonal injur*”[tiab] OR post-concuss*[tiab]). Full search strategies for each database are reported verbatim in Supplementary Appendix A.

The inclusion criteria encompassed studies that utilized TBI (i.e., human or animal) as a model of neurological dysfunction in the context of ketogenic therapy as an intervention. This includes studies involving the ketogenic diet, exogenous ketone supplements, and/or interventions that induce ketosis. Studies were included if they reported original quantitative outcomes (clinical, functional, or metabolic). Clinical studies were only included if they enrolled more than five patients. This threshold was applied to exclude very small case series, which lack the methodological structure to permit meaningful characterization of interventions and outcomes at the level required for this evidence map. Studies that were performed *in vitro* or using isolated cells, not involving TBI or ketogenic diet in any form, were excluded. Non-English publications, reviews, duplicate reports, editorials, letters, commentaries, and conference abstracts were also excluded. This decision was pragmatic, reflecting translation resource constraints of the review team. Full diagrams encompassing inclusion and exclusion criterias are provided in Supplementary Appendix B.

Following database searches, all retrieved records were imported into Rayyan, a web-based review management platform (17). Duplicates were identified and removed using platform’s automated deduplication function, supplemented by manual review by a senior reviewer (Author 2). Two reviewers (Author 1 and Author 2) independently screened titles and abstracts identified through the literature search. Abstracts deemed eligible by both reviewers were included in the final phase of the screening process. Discrepancies and conflicts were resolved by another reviewer (Author 3). Full-text versions of the eligible studies were reviewed and analyzed for data extraction.

### Data extraction

Data extraction captured key variables from each included study, including the title, author, year, country, type of study, model, population, sex, age group, injury model, type of intervention(s), intervention details, outcome(s) evaluated, overall effect(s), primary mechanism(s) studied, and relevance to review.

Human studies were evaluated across three primary outcome domains: feasibility (achievement of ketosis, ketone utilization, and metabolic stability), safety (adverse clinical events and participant tolerance), and functional efficacy (neurological recovery, cognitive performance, and energy intake). Animal studies were similarly evaluated across three domains: metabolic outcomes (cerebral energy production, mitochondrial function, oxidative stress, and secondary injury signaling), neuroprotective outcomes (structural brain integrity, neuroinflammation, and neurovascular function), and behavioral outcomes (motor performance, cognition, and affective behavior).

## Results

The initial search yielded 421 studies, 116 (27.55%) of which were duplicates. 242 (57% of the initial pool) studies were excluded according to their titles, abstracts, and relevance to this review. 36 (8.5% of the initial pool) full-text papers were evaluated based on established inclusion and exclusion criteria. 28 (6.6% of the initial pool) papers were determined to meet this review’s inclusion criteria. 4 studies were added manually at the end of screening full-text papers. A complete list of studies excluded at full-text review stage with reasons for exclusion is provided in Supplementary Appendix C (see References (18–25)). The complete screening process is illustrated in a PRISMA flowchart (Supplementary Appendix D).

The geographical distribution of studies revealed a predominance from the United States, accounting for 62.50% (*n* = 20) of the total. China accounts for 15.63% (*n* = 5), while Australia accounts for 6.25% (*n* = 2) of the total number of studies. Canada, the United Kingdom, Turkey, Israel, Poland, and Switzerland each accounted for 3.13% (*n* = 1) of the total number of studies. 25 out of 32 (78.13%) studies were conducted using animal models, while 7 (21.88%) were human clinical trials.

### Human studies

Clinical trials enrolled patients with acute brain injury (57.14%, *n* = 4), TBI (28.57%, *n* = 2), and post-concussion syndrome (14.29%, *n* = 1) (Table 1). Ketogenic interventions included dietary administration and enteral nutrition, with 42.86% (*n* = 3) utilizing ketone body supplementation via enteral or nasogastric routes. Only one trial utilized glucose infusion (14.29%, *n* = 1). Most studies (71.43%, *n* = 5) initiated intervention immediately post-injury, while 28.57% (*n* = 2) implemented delayed intervention protocols.

**Table 1:** This table summarizes the primary aim of clinical trials and the corresponding outcome of ketogenic intervention during TBI recovery.

Author and year	Title	Injury type	Outcome
Rippee et al. 2020 (32)	The ketogenic diet in the treatment of post-concussion syndrome—a feasibility study	PCS	This clinical trial assessed the feasibility and safety of the very high-fat ketogenic diet in adult patients suffering from post-concussion syndrome. Visual memory improved by a mean of 12.2 points on ImPACT, PCSS scores trended down at month 2 but were not significant, and ketosis was achieved in 79% of participants on 80% of intervention days.
Bernini et al. 2020 (33)	Modulation of cerebral ketone metabolism following traumatic brain injury in humans	TBI	This research study was able to track cerebral metabolism in adults after brain injury. Fasting produced 2.8-fold higher brain interstitial ketone bodies vs. fed state, MCT feeding elevated plasma C8 and brain C8/C10, and brain and plasma βHB were positively correlated.
Arora et al. 2022 (34)	Phase I single center trial of ketogenic diet for adults with traumatic brain injury	TBI	This study evaluated clinical feasibility as well as metabolic outcomes in patients with traumatic brain injuries treated with ketogenic diet. Ketosis was achieved in 8 of 10 patients with glucose declining ~3.9 mg/dL/day. Two patients developed hypertriglyceridemia. No metabolic acidosis or organ injury occurred. Neurological outcomes were mixed.
Edwards et al. 2024 (35)	Diet-induced ketosis in adult patients with subacute acquired brain injury: a feasibility study	ABI	This study evaluated clinical outcomes and feasibility in patients with traumatic brain injuries treated with ketogenic diet. Ketosis was achieved within a median of 1 day and maintained 97% of the time. HbA1c decreased in 92% of patients. Functional recovery scores were relatively unchanged upon admission versus four weeks. Hyponatraemia occurred in 3 patients.
White et al. 2020 (36)	Inducing ketogenesis via an enteral formulation in patients with acute brain injury: a phase II study	ABI	This paper assessed ketogenic intervention and its safety in patients with varying degrees of acute brain injury. 90% of patients raised plasma βHB. Serum glucose fell from 8.7 to 7.0 mmol/L by day 2. Lactate normalized below 1.0 mmol/L. Liver enzymes rose over 6 days. One metabolic acidosis case was recorded.
Ritter et al. 1996 (37)	Evaluation of a carbohydrate-free diet for patients with severe head injury	ABI	This clinical trial evaluated carbohydrate restriction in patients with severe brain injury. βHB was elevated and pyruvate reduced in the KD group. Plasma triglycerides were significantly higher from days 5–14. Urinary nitrogen balance improved. GCS recovery rate was not significantly different between groups.
Robertson et al. 1991 (31)	The effect of glucose administration on carbohydrate metabolism after head injury	ABI	This clinical evaluated the effects of intravenous glucose administration on patients with severe head injury. IV glucose elevated insulin and suppressed ketogenesis. CMR of ketone bodies increased in the saline group. High arterial glucose correlated with poor neurological recovery and mortality.

Outcomes reflect direction and overall impact within clinical contexts. The table includes the methodology used to induce TBI and the types of TBI used in the human studies included in this review. (PCS; Post-Concussion Syndrome, TBI; Traumatic Brain Injury, ABI; Acute Brain Injury, ImPACT; Immediate Post-Concussion Assessment and Cognitive Testing, PCSS; Post-Concussion Symptom Scale, MCT; Medium-Chain Triglycerides, C8; Caprylic Acid, C10; Capric Acid, *β*HB; Beta-Hydroxybutyrate, HbA1c; Glycated Hemoglobin, KD; Ketogenic Diet, GCS; Glasgow Coma Scale, IV; Intravenous, CMR; Cerebral Metabolic Rate).

Outcome assessments were categorized into three primary domains. Feasibility was defined as the extent to which a clinical trial or intervention could be successfully implemented in terms of participant recruitment, protocol adherence, retention rates, safety, and resource requirements. There were several measures for feasibility, including achievement of ketosis, utilization of ketone bodies, and metabolic stability, were evaluated in all studies utilizing laboratory assessments (100%, *n* = 7). Safety outcomes, encompassing adverse clinical events and patient tolerance, were assessed in 71.43% (*n* = 5) and 57.14% (*n* = 4) of studies, respectively. Clinical efficacy measures were limited, with neurological recovery evaluated in 57.14% (*n* = 4) of studies, energy intake in 28.57% (*n* = 2), and cognitive performance in only 28.57% (*n* = 2). Overall, clinical investigations demonstrated feasibility in 85.71% (*n* = 6) and safety of ketogenic interventions in 71.43% (*n* = 5). No studies provided adequate evidence of long-term clinical efficacy.

### Non-human studies

Rats were the most common experimental model, with 76% (*n* = 19) of animal studies using either Sprague Dawley Rats or Wistar Rats. Adult models were the primary developmental stage of choice across the studies. 72% (*n* = 18) of animal studies were conducted in adult animal models, though rats, mice, and flies were found to have ranged from adolescent, juvenile, and newly eclosed across the included animal studies. Notably, 68% (*n* = 17) studies reported no utilization female specimens, indicating a strong predominance of male cohorts.

TBI was mainly induced in animals through cortical contusion injury (CCI) techniques. 68% (*n* = 17) of studies utilized CCI, while closed head trauma (CHT) and fluid percussion injury (FPI) were utilized less frequently (Table 2). Ketogenic interventions were primarily delivered through dietary interventions. 68% (*n* = 17) of animal studies utilized the ketogenic diet, while additional modes of intervention included ketone-ester supplementation, ketone body supplementation, and ketone body infusion. Only one study utilized an acetate precursor. 80% (*n* = 20) of animal studies initiated ketogenic intervention immediately after injury, while 20% (*n* = 5) of animal studies utilized ketogenic intervention in cohorts before and after injury.

**Table 2:** This table summarizes the primary aim of preclinical studies and the corresponding outcome of ketogenic intervention during TBI recovery.

Author and year	Title	Technique	Injury type	Outcome
Arun et al. 2010 (46)	Metabolic acetate therapy for the treatment of traumatic brain injury	CCI device	CCI	This study utilized GTA administration in rats suffering from brain injury. Acetate precursor administration in rats significantly improved NAA and ATP production, and locomotor performance on rotarod significantly improved vs. untreated groups
Greco et al. 2016 (47)	Ketogenic diet decreases oxidative stress and improves mitochondrial respiratory complex activity	Electronically controlled pneumatic piston cylinder	CCI	These researchers evaluated mitochondrial stress in rats by inducing them with a CCI and treating them with the ketogenic diet. KD reduced oxidative markers 3-NT and 4-HNE, elevated NQO1, SOD1/2, and partially restored mitochondrial complex II/III activity, though complex I was not rescued.
Dilimulati et al. 2023 (48)	Ketogenic diet modulates neuroinflammation via metabolites from *Lactobacillus reuteri* after repetitive mild traumatic brain injury in adolescent mice	PinPoint PCI3000 precision control impactor)	CCI	This study focused on highlighting the effects of the ketogenic diet on neuroinflammation during TBI recovery in mice. KD elevated serum and brain βHB, reduced TNF-α and IL-1β, decreased Iba-1 + and GFAP+ cell counts, and improved beam walk and Y-maze performance.
Mu et al. 2022 (49)	Ketogenic diet protects myelin and axons in diffuse axonal injury	Marmarou method	DAI	This investigation studied the neuroprotective effects of the ketogenic diet in rats in rats with diffuse axonal injury. KD elevated βHB, ATP, and NAD + within 3 days, preserved myelin integrity, reduced Drp1-mediated mitochondrial fission, and decreased APP and p-NF-H axonal injury markers.
Har-Even et al. 2021 (44)	Ketogenic diet as a potential treatment for traumatic brain injury in mice	CCI device	CHT	These researchers evaluated the effects of the ketogenic diet following traumatic brain injury in mice. KD restored SIRT1 expression and preserved NeuN+ neurons in the cortex and dentate gyrus, reduced GFAP in the dentate gyrus, and improved novel object recognition and Y-maze, though anxiety was unchanged.
Almeida-Suhett et al. 2022 (50)	The Ketone Ester, 3-hydroxybutyl-3-hydroxybutyrate, attenuates neurobehavioral deficits and improves neuropathology following controlled cortical impact in male rats	CCI device	CCI	This study examined the benefits of ketone supplements as a source of alternative energy in rats following brain injury. KE gavage elevated blood βHB, reduced lesion volume and GFAP+ staining across all brain regions, decreased Iba-1 + in cortex and hippocampus, and improved NSS-R sensory scores.
Hu et al. 2009 (51)	The protective effect of the ketogenic diet on traumatic brain injury-induced cell death in juvenile rats”	CCI device	CCI	This paper investigated the age-dependent effects of the ketogenic diet in rats with TBI. KD elevated βHB, reduced pro-apoptotic Bax expression and TUNEL+ cells in the penumbra, and decreased brain water content, indicating reduced edema.
Prins et al. 2009 (52)	The Effects of age and ketogenic diet on local cerebral metabolic rates of glucose after controlled cortical impact injury in rats	Electronically controlled pneumatic piston cylinder	CCI	These researchers evaluated cerebral metabolism in the brains of rats administered a ketogenic diet across different phases of life. Ketogenic intervention elevated βHB and restored CMRglc to sham levels by day 7 in PND70 rats, while contusion volume was reduced 31% in PND35 only, with no benefit in aged animals.
Zhang et al. 2018 (41)	Proton magnetic resonance spectroscopy (H1-MRS) study of the ketogenic diet on repetitive mild traumatic brain injury in adolescent rats and its effect on neurodegeneration	Fluid pressure pulse	FPI	These researchers were able to study the effects of the ketogenic diet in rats with mild traumatic brain injury. KD elevated βHB and the NAA/Cr ratio and upregulated beclin-1, indicating improved autophagic flux, while no tissue loss was detected and beam balance latency was decreased.
Prins et al. 2004 (53)	Increased cerebral uptake and oxidation of exogenous βHB improves ATP following traumatic brain injury in adult rats	Electronically controlled pneumatic piston cylinder	CCI	These researchers investigated mechanisms underlying cerebral metabolism and its relationship with the ketogenic diet after traumatic brain injuries. βHB infusion produced an 8.5-fold increase in cerebral uptake and a 10.7-fold rise in CO2 production post-CCI, with cortical ATP levels fully restored.
Orhan et al. 2016 (43)	Effects of beta-hydroxybutyrate on brain vascular permeability in rats with traumatic brain injury	Lateral fluid percussion	FPI	This study looked at βHB and its effects on the blood–brain barrier integrity after traumatic brain injury in rats. βHB reduced GSH and MDA and partially decreased HRP extravasation, but failed to restore GFAP immunoreactivity, while tight junction ultrastructure remained intact across all groups.
Thau-Zuchman et al. 2021 (54)	A new ketogenic formulation improves functional outcome and reduces tissue loss following traumatic brain injury in adult mice	PCI3000 precision cortical impactor	CCI	This study assessed a novel ketogenic formulation in mice following ketogenic therapy. The new KD formulation elevated βHB and p-mTOR, reduced lesion size and microglial activation, increased oligodendrocyte regeneration, and improved mNSS, rotarod, and water maze performance.
Salberg et al. 2019 (39)	The behavioral and pathophysiological effects of the ketogenic diet on mild traumatic brain injury in adolescent rats	Lateral impact technique	CHT	These researchers studied the pathophysiological effects of the ketogenic diet. KD elevated ketones and Opa1, reduced Fgf2 and Iba1 in mTBI animals, improved intestinal tight junction gene expression, and reduced forced swim immobility and EPM anxiety, with sex-dependent Sirt1 effects.
Hu et al. 2009 (55)	Ketogenic diet reduces cytochrome-crelease and cellular apoptosis following traumatic brain injury in juvenile rats	CCI device	CCI	This paper studied the pathology of the ketogenic diet and how it affects mitochondrial functionality after a traumatic brain injury. KD elevated βHB, reduced cytosolic cytochrome-c release, decreased caspase-3 and TUNEL+ apoptotic cells in the penumbra, and reduced brain edema.
Greco et al. 2020 (38)	Alternative substrate metabolism depends on cerebral metabolic state following traumatic brain injury	Electronically controlled pneumatic piston cylinder	CCI	This investigation looked at how alternative energy substrates that would improve cerebral metabolism and energy utilization between male and female animals. In males, βHB administration increased state 3 respiration and acetyl-CoA, while in females early βHB worsened RCR and reduced ATP, demonstrating sex and timing-dependent mitochondrial effects.
Deng-Bryant et al. 2011 (56)	Ketogenic diet prevents alterations in brain metabolism in young but not adult rats after traumatic brain injury	Electronically controlled pneumatic piston cylinder	CCI	These researchers tracked age-related effects of the ketogenic diet after traumatic brain injury in rats. KD restored ATP, PCr/Cr, NAA, and GABA toward sham levels at 24 h in PND35 rats, while no significant effect on brain energy metabolites was observed in PND70 animals.
Scafidi et al. 2022 (57)	Metabolism of exogenous [2,4-13C] β-hydroxybutyrate following traumatic brain injury in 21-22-Day-old rats: an *Ex Vivo* NMR study	CCI device	CCI	These researchers tracked cerebral ketone utilization after traumatic brain injury in rats administered exogenous ketone body infusion. ^13^C-βHB tracing confirmed active cerebral TCA cycle oxidation post-injury, with increased label incorporation into lactate bilaterally, indicating unimpaired ketone utilization after CCI.
Appelberg et al. 2009 (25)	The effects of a ketogenic diet on behavioral outcome after controlled cortical impact injury in the juvenile and adult rat	CCI device	CCI	This paper analyzed the behavioral outcomes of ketogenic therapy in rats with traumatic brain injury. KD elevated βHB and reduced glucose and lactate. PND35 rats showed preserved beam walk and water maze performance, while adult rats demonstrated no functional benefit from KD.
Prins et al. 2005 (58)	Age-dependent reduction of cortical contusion volume by ketones after traumatic brain injury	Electronically controlled pneumatic piston cylinder	CCI	This study evaluated the age-related effects of ketone supplementation after traumatic brain injury. KD elevated βHB and reduced cortical contusion volume and Fluoro-Jade+ degenerating cells in PND35 and PND45 rats, with a lower βHB dose producing greater tissue sparing than a higher dose.
Davis et al. 2008 (59)	Fasting is neuroprotective following traumatic brain injury	CCI device	CCI	This paper investigated fasting-induced ketogenic diet effects on rats following TBI. 24 h fasting reduced mitochondrial ROS, calcium loading, and lipid peroxidation, increased state III ADP utilization, improved cortical tissue sparing, and enhanced water maze cognition, while swim speed was unchanged.
Kawon et al. 2026 (40)	Modulatory role of the ketogenic diet in glial scar formation after traumatic brain injury: a Fourier transform infrared, Raman, and X-ray fluorescence microscopy study	CCI device	CCI	Kawon et al. examined the effects of the ketogenic diet on glial scar formation. Overall, this study highlights that the ketogenic diet modulates brain tissue remodeling after injury in a sex-dependent manner, underscoring the importance of considering sex and timing when evaluating ketogenic intervention as a therapeutic strategy for traumatic brain injury.
Schwartzkroin et al. 2010 (42)	Does ketogenic diet alter seizure sensitivity and cell loss following fluid percussion injury?	Fluid percussion	FPI	This study evaluated the effects of the ketogenic diet on seizures and apoptosis following traumatic brain injury. KD elevated βHB but had no effect on hippocampal cell loss, astrogliosis, or microgliosis, while seizure latency to myoclonic jerk and bilateral forelimb clonus stages was increased.
Rimkus et al. 2025 (60)	Traumatic brain injury reprograms lipid droplet metabolism shaped by aging and diet in drosophila brain	HIT device	CHT	This paper examined lipid metabolism following traumatic brain injury in flies. KD altered lipid droplet size and number post-TBI, with accumulated LDs interpreted as stored substrate for β-oxidation, and early post-TBI mortality was reduced in KD-fed flies.
Lee et al. 2019 (45)	Dietary supplementation with the ketogenic diet metabolite beta-hydroxybutyrate ameliorates post-tbi aggression in young-adult male drosophila	Variant of HIT device	CHT	This paper evaluated affective and behavioral outcomes of flies with TBI placed on ketogenic intervention. βHB supplementation significantly reduced post-TBI aggression in male flies via KATP channel modulation, while lifespan, MI24, and female behavior were unaffected.
Blommer et al. 2021 (61)	Ketogenic diet reduces early mortality following traumatic brain injury in drosophila via the PPARγ ortholog Eip75B	HIT device	CHT	Researchers evaluated molecular mechanisms related to lifespans of flies with TBI under the ketogenic diet. KD activated the PPARγ ortholog Eip75B, reducing early post-TBI mortality and extending median lifespan in injured flies, while MI24 was equivalent to water-fed controls.

Outcomes reflect direction and overall impact within experimental contexts. The table includes the methodology used to induce TBI and the types of TBI used in the animal included in this review. (CCI; Controlled Cortical Impact, DAI; Diffuse Axonal Injury, CHT; Closed Head Trauma, FPI; Fluid Percussion Injury, HIT; High-Impact Trauma, KD; Ketogenic Diet, KE; Ketone Ester, GTA; Glyceryl Triacetate, *β*HB; Beta-Hydroxybutyrate, NAA; N-Acetylaspartate, Cr; Creatine, ATP; Adenosine Triphosphate, NAD+; Nicotinamide Adenine Dinucleotide, ADP; Adenosine Diphosphate, CoA; Coenzyme A, CO2; Carbon Dioxide, TCA; Tricarboxylic Acid, PCr/Cr; Phosphocreatine/Creatine, GABA; Gamma-Aminobutyric Acid, CMRglc; Cerebral Metabolic Rate of Glucose, RCR; Respiratory Control Ratio, ROS; Reactive Oxygen Species, 3-NT; 3-Nitrotyrosine, 4-HNE; 4-Hydroxynonenal, NQO1; NAD(P)H Quinone Oxidoreductase 1, SOD1/2; Superoxide Dismutase 1/2, TNF-α; Tumor Necrosis Factor Alpha, IL-1β; Interleukin-1 Beta, Iba-1; Ionized Calcium-Binding Adapter Molecule 1, GFAP; Glial Fibrillary Acidic Protein, APP; Amyloid Precursor Protein, p-NF-H; Phosphorylated Neurofilament Heavy Chain, Drp1; Dynamin-Related Protein 1, SIRT1; Sirtuin 1, NeuN; Neuronal Nuclei, Bax; BCL2-Associated X Protein, TUNEL; Terminal Deoxynucleotidyl Transferase dUTP Nick End Labeling, GSH; Glutathione, MDA; Malondialdehyde, HRP; Horseradish Peroxidase, p-mTOR; Phosphorylated Mechanistic Target of Rapamycin, mNSS; Modified Neurological Severity Score, NSS-R; Neurological Severity Score-Revised, EPM; Elevated Plus Maze, Opa1; Optic Atrophy 1, Fgf2; Fibroblast Growth Factor 2, PPARγ; Peroxisome Proliferator-Activated Receptor Gamma, Eip75B; Ecdysone-Induced Protein 75B, KATP; ATP-Sensitive Potassium Channel, LD; Lipid Droplet, MI24; Mortality Index at 24 Hours, mTBI; Mild Traumatic Brain Injury, NMR; Nuclear Magnetic Resonance, ^**1**^H-MRS; Proton Magnetic Resonance Spectroscopy, PND; Postnatal Day).

Study outcomes were evaluated using clusters of frameworks most assessed in non-human studies. The restoration of energy metabolism following TBI was assessed using biochemical and molecular biomarkers in 80% of studies (*n* = 20), while mitochondrial dynamics encompassed measures of mitochondrial respiration, efficiency, and oxidative stress in 28% (*n* = 7) of studies. While measurements of secondary injury signaling pathways were assessed in 40% (*n* = 10) of studies, assessment of apoptosis, often through markers such as Bax/Bcl-2 expression and caspase activation, was conducted in only 8% (*n* = 2) of studies. Tissue loss and neurodegeneration were measured across 36% (*n* = 9) and 24% (*n* = 6) of studies, respectively. Measures of neuroinflammation were assessed via inflammatory and glial biomarkers in 28% (*n* = 7) of studies. Neurovascular integrity, which assessed blood–brain barrier integrity and vascular permeability, was only evaluated in 16% (*n* = 4) of studies. Mobility was assessed in 28% (*n* = 7) of studies utilizing motor performance tasks, while only 12% (*n* = 3) of studies evaluated memory and cognitive task performance following administration of ketogenic intervention. Affective behaviors, including measures of mood and anxiety, were also only mentioned in 12% (*n* = 3) of studies. Global outcomes, reflecting survival-related measures, were assessed in 20% (*n* = 5) of studies, despite primarily occurring in experiments utilizing *Drosophila*. Overall, animal experiments demonstrated positive effects on metabolic pathways in 22 out of 25 (92.00%) studies, positive neuroprotective effects in 12 out of 25 (48.00%) studies, and positive behavioral improvements in 13 out of 25 (52.00%), (Table 3 A–C).

**Table 3:** Frameworks for animal studies were evaluated across domains of cellular and metabolic pathology (A), neuroprotective effects (B), and behavioral assays (C).

(A) Author and year	Metabolic rescue	Mitochondrial dynamics	Apoptosis	Secondary injury signaling
Arun et al. 2010 (46)	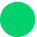			
Greco et al. 2020 (38)		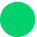		
Dilimulati et al. 2023 (48)	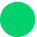			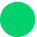
Mu et al. 2022 (49)	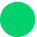	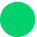		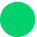
Har-Even et al. 2021 (44)	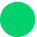			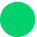
Almeida-Suhett et al. 2022 (50)	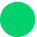			
Hu et al. 2009 (51)	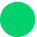		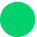	
Prins et al. 2009 (52)	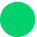			
Zhang et al. 2018 (41)	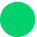			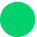
Prins et al. 2004 (53)	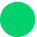			
Orhan et al. 2015 (43)	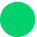	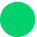		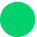
Thau-Zuchman et al. 2021 (54)	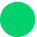	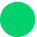		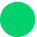
Salberg et al. 2019 (39)	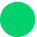	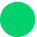		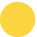
Hu et al. 2009 (51)	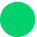		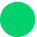	
Greco et al. 2020 (38)	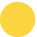	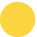		
Deng-Bryant et al. 2011 (56)	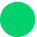			
Scafidi et al. 2022 (57)	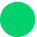			
Appelberg et al. 2009 (25)	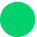			
Prins et al. 2005 (58)	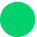			
Davis et al. 2008 (59)		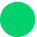		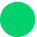
Kawon et al. 2026 (40)	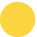			
Schwartzkroin et al. 2010 (42)				
Rimkus et al. 2025 (60)				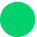
Lee et al. 2019 (45)				
Blommer et al. 2021 (61)	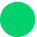			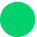

(B) Author and year	Tissue loss	Neurodegeneration	Neuroinflammation	Neurovascular integrity
Arun et al. 2010 (46)				
Greco et al. 2016 (38)				
Dilimulati et al. 2023 (48)		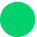		
Mu et al. 2022 (49)		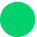		
Har-Even et al. 2021 (44)		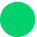	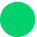	
Almeida-Suhett et al. 2022 (50)	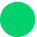	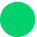	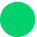	
Hu et al. 2009 (51)	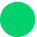			
Prins et al. 2009 (52)	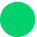			
Zhang et al. 2018 (41)	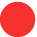			
Prins et al. 2004 (53)				
Orhan et al. 2015 (43)			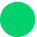	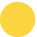
Thau-Zuchman et al. 2021 (54)	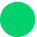		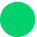	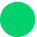
Salberg et al. 2019 (39)			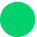	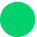
Hu et al. 2009 (51)	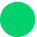			
Greco et al. 2020 (38)				
Deng-Bryant et al. 2011 (56)				
Scafidi et al. 2022 (57)				
Appelberg et al. 2009 (25)				
Prins et al. 2005 (58)	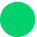	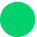		
Davis et al. 2008 (59)	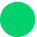			
Kawon et al. 2026 (40)			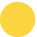	
Schwartzkroin et al. 2010 (42)	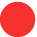	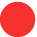	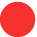	
Rimkus et al. 2025 (60)				
Lee et al. 2019 (45)				
Blommer et al. 2021 (61)				

(C) Author and year	Mobility	Memory	Mood	Global outcomes
Arun et al. 2010 (46)	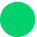			
Greco et al. 2016 (47)				
Dilimulati et al. 2023 (48)	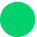	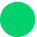		
Mu et al. 2022 (49)				
Har-Even et al. 2021 (44)	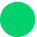		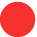	
Almeida-Suhett et al. 2022 (50)				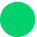
Hu et al. 2009 (51)				
Prins et al. 2009 (52)				
Zhang et al. 2018 (41)	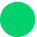			
Prins et al. 2004 (53)				
Orhan et al. 2016 (43)				
Thau-Zuchman et al. 2021 (54)	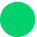	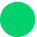		
Salberg et al. 2019 (39)			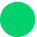	
Hu et al. 2009 (51)				
Greco et al. 2020 (38)				
Deng-Bryant et al. 2011 (56)				
Scafidi et al. 2022 (57)				
Appelberg et al. 2009 (25)	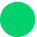			
Prins et al. 2005 (52)				
Davis et al. 2008 (59)	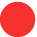	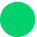		
Kawon et al. 2026 (40)				
Schwartzkroin et al. 2010 (42)				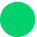
Rimkus et al. 2025 (60)				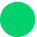
Lee et al. 2019 (45)			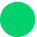	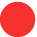
Blommer et al. 2021 (61)				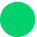

Outcomes were classified as positive (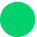), mixed (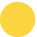), and negative (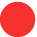). Outcomes that reflected null results despite evaluations performed were classified as neutral (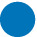). Blank cells indicate that the specific domain was not evaluated in the corresponding study.

## Discussion

This study synthesizes evidence from the literature highlighting the therapeutic effects of ketogenic interventions in various areas of TBI. The literature was systematically reviewed and analyzed, demonstrating growing evidence of the benefits of ketogenic therapy in preclinical studies.

### Overview

Post-injury pathology following TBI stems from the disruption of the brain’s reliance on glucose as a primary energy substrate (26, 27). Under conditions of energetic stress and relative hypoxia, often induced by TBI, mitochondrial respiration becomes impaired, resulting in reduced aerobic ATP production (28, 29). When sustained, anaerobic glycolysis can contribute to chronic bioenergetic deficiency within the brain (7, 29). Because alternative substrates for cerebral energy production during metabolic crises are limited, ketone bodies serve as an important compensatory fuel during periods of impaired glucose utilization. Ketone bodies such as *β*-hydroxybutyrate (βHB) can be transported across the blood–brain barrier via monocarboxylate transporters and may be derived from endogenous fatty acid oxidation or exogenous supplementation of βHB (27, 30). Within neurons, βHB is converted to acetoacetate, which is cleaved to form acetoacetyl-CoA to enter the citric acid cycle and support ATP production (27, 29, 30). Improved energy availability may also contribute to more effective treatments of secondary injury processes associated with TBI (26, 27). The experimental and clinical findings synthesized in this review are largely consistent within this biological framework.

### Clinical perspectives for ketogenic intervention in traumatic brain injury

Interest in ketogenic strategies has its roots in early metabolic observations. In 1991, Robertson et al. reported that comatose patients with ABI (*n* = 21) receiving intravenous glucose demonstrated increased cerebral lactate production and reduced ketone body uptake compared with controls, suggesting that exogenous glucose may impair the brain’s capacity to utilize ketone bodies following injury (31). These findings served as among the first to underscore the potential of ketone metabolism as a therapeutic strategy for TBI. Notably, critically ill patients with acute brain injury were able to reach ketosis under monitored clinical conditions through diet, enteral, and nasogastric administration at a similar rate to cohorts of patients afflicted with post-concussion syndrome and TBI (31–36). This suggests that the brain can engage in ketone metabolism in acute conditions. Despite high levels of ketosis achievement, adherence throughout the studies included in this review varied, with several papers citing metabolic complications. For example, Arora et al. examined 10 patients with TBI administered a 4:1 ketogenic diet, reporting a single case of hypoglycemia because of ketogenic intervention (34). The event was resolved with dextrose infusion without recurrence, and the patient subsequently tolerated ketogenic therapy for the rest of the investigation. In another investigation, Edwards et al. examined 29 patients who received ketone body supplements and/or a 2.5:1 ketogenic diet if they were capable of oral intake, reporting electrolyte instability as an adverse outcome (35). Within this study, 3 of 12 patients developed hyponatremia (<135 mmol/L) during the intervention period. Only one out of the three patients were excluded due to severe hyponatremia (122 mmol/L).

Gastrointestinal problems were one of the most common complications (32, 35–37). Rippee et al. examined 14 patients suffering from post-concussion syndrome given a very high-fat ketogenic diet, reporting diarrhea in one patient and nausea in another (32). No patient noted any tolerability issues during a follow up survey (32). White et al. enrolled 20 patients with acute brain injury who received a 4:1 ketogenic supplementation regimen via nasogastric tube and documented 8 episodes of diarrhea or vomiting during the intervention period (36). In a separate study, Ritter et al. had to dilute the full strength ketogenic diet because of the high frequency of diarrhea it resulted in the acute brain injury patients (*n* = 20) they studied, highlighting the effects of variable formulation of ketogenic intervention may have on patients (37).

Weight and energy intake were relatively well maintained across studies, indicating that the ketogenic intervention did not compromise nutritional delivery (31, 34, 35). While Robertson et al. indicated a higher caloric intake across the 5-day study period within the glucose treatment group, Rippee et al. highlighted that energy intake between the control group and the experimental group was similar (31, 32). Similarly, Edwards et al. noted no significant difference in energy intake between experimental groups and control groups, despite one patient being excluded due to loss of appetite and challenges in consumption (35).

Only a limited number of studies were able to evaluate the clinical efficacy of ketogenic intervention (32, 34, 35, 37). Rippee et al. noted that while the post-concussion syndrome scale (PCSS) trended toward improvement from baseline at month 2, the data did not reach statistical significance (32). Visual memory was assessed by the ImPACT (Immediate Post-Concussion Assessment and Cognitive Testing) tool, which improved significantly among compliant participants, with a mean improvement of 12.2 points (*p* = 0.02). Notably, participants who did not reach ketosis were not able to achieve these improvements and, in some cases, worsened scores. In cohorts with more severe injury, neurological recovery metrics were unchanged. Arora et al. presented heterogeneous trajectories, noting that one patient continued to have poor neurological exams before the family decided to withdraw care, while another recovered neurologically within four days (35). Ritter et al. found that GCS scores improved during the 2 weeks of the study between the control group and the experimental group, though overall recovery metrics did not show any significant difference (37).

### Preclinical evidence on ketogenic intervention following traumatic brain injury

Multiple preclinical studies demonstrated that ketogenic intervention is useful for the energetic crisis caused by TBI. The rapid shift in substrate utilization is characterized by elevated ketone availability and the restoration of ATP-related biomarkers. This interaction subsequently improves mitochondrial functionality, augmenting respiratory efficiency, and reducing oxidative stress. However, several studies highlighted the sex, age, and injury dependent effects that ketogenic intervention has on various aspects of metabolic rescue (38–40). While ketogenic therapy can restore bioenergetic dysfunction, its influence on molecular signaling and metabolism could potentially be context dependent.

Ketogenic intervention was associated with significant reduction in tissue loss and neurodegeneration in preclinical studies. However, studies using fluid percussive techniques in rats reported no observable histological abnormalities, no significant differences in hippocampal tissue loss, and persistent astrocyte disruption and blood–brain barrier compromise in both ketogenic and control groups, suggesting that ketogenic interventions were unable to attenuate pathological processes in isolation (41–43). Notably, the study reporting the most pronounced null findings was the only one in this review to utilize an exclusively female cohort, further implicating sex as a potential moderator of neuroprotective outcomes (43). These patterns support context dependent effects of the ketogenic diet by demonstrating that age, sex, and injury severity may have an influence on the neuroprotective efficiency of ketogenic therapy in animals.

While mobility outcomes showed stronger effects across studies, cognitive evaluations were relatively underpowered despite the positive findings. Additionally, affective behavior and global evaluations were less frequently utilized, yielding much weaker results. Studies examining affective behavior produced mixed results, with some CHT models showing no significant effects while others demonstrated promising improvements (39, 44, 45). Global outcome measures were predominantly carried out in *Drosophila* models, where ketogenic interventions were able to reduce early mortality, though lifespan extension was not altered significantly (45). Selection of experimental models, assessment frequency, and behavioral paradigms could potentially shape preclinical outcomes that define efficiency of ketogenic therapy in functional recovery after TBI.

### Limitations, gaps in literature, and future considerations

Substantial methodological heterogeneity encompassing differences in injury models, ketogenic formulation, timing of intervention, species, age, and outcome measures limits direct cross-study comparison when interpreting and synthesizing the findings of this study. We acknowledge the absence of registration due to the nature of this review as a potential source of bias at the review level.

Future clinical trials should adopt standardized outcome reporting frameworks encompassing functional independence measures at extended follow up timepoints of at least 6 and 12 months post-injury. Future investigations should also examine how ketogenic interventions perform across the lifespan, given that metabolic responses to both TBI and dietary modification are known to vary with age. Although a small number of animal studies using female models reported sex dependent effects of ketogenic therapy, majority of clinical trials conducted relied exclusively on male dominated cohorts. Given the well documented influence of sex hormones on neuroinflammation, metabolism, and TBI recovery trajectories, future studies should be designed with sufficient power to detect and characterize sex specific responses to ketogenic interventions.

## Conclusion

This scoping review synthesizes the current state of ketogenic interventions aiding in post-TBI recovery efforts. The studies demonstrated a consistent improvement in metabolic and cellular outcomes in animal models, while neuroprotective and functional effects remained to be variable. Substantial methodological heterogeneity limits clinical translation. Clinical studies were able to confirm feasibility and safety but lacked adequate sampling power to establish efficacy. Larger, standardized trials are needed to determine whether ketogenic therapy improves long term patient outcomes following TBI.
