# Supplementary material for: Administration of ketogenic intervention as a potential treatment during post-traumatic brain injury recovery: a scoping review
Source: Front Nutr. 2026 May 29;13:1848682. doi: 10.3389/fnut.2026.1848682 (PMC13261819; doi:10.3389/fnut.2026.1848682)
Supplement: Supplementary file 1 [file Table_1.docx]

**Table 1A.** Full search strategy for PubMed

| **Set #** | **PubMed** |
| --- | --- |
| **#1 Ketogenic Diet** | *("Diet, Ketogenic"[Mesh] OR ketogen*[tiab] OR "ketogenic diet"[tiab] OR ketosis[tiab] OR "Ketone Bodies"[Mesh] OR keton*[tiab])* |
| **#2 TBI** | *("Brain Injuries, Traumatic"[Mesh] OR "Craniocerebral Trauma" [Mesh] OR "Concussion" OR (traumatic[tiab] AND brain injur*[tiab]) OR head injur*[tiab] OR TBI[tiab] OR mTBI[tiab] OR concuss*[tiab] OR "diffuse axonal injur*"[tiab] OR post-concuss*[tiab])* |
| **#3** | #1 AND #2 |

**Table 1B.** Full search strategy for Web of Science

| **Set #** | **Web of Science** |
| --- | --- |
| **#1 Ketogenic Diet** | (ketogen* OR "ketogenic diet" OR ketosis OR keton*) |
| **#2 TBI** | ("brain injur*" OR "head injur*" OR TBI OR mTBI OR concussion OR concuss* OR "diffuse axonal injur*" OR post-concuss* OR "traumatic brain injur*") |
| **#3** | #1 AND #2 |
