# Supplementary material for: Administration of ketogenic intervention as a potential treatment during post-traumatic brain injury recovery: a scoping review
Source: Front Nutr. 2026 May 29;13:1848682. doi: 10.3389/fnut.2026.1848682 (PMC13261819; doi:10.3389/fnut.2026.1848682)
Supplement: Supplementary file 2 [file Table_2.docx]

**Supplementary Appendix B**

**Inclusion Criteria**

1. Clinical or preclinical studies investigating ketogenic-based interventions in traumatic brain injury
2. Study designs including randomized controlled trials, non-randomized clinical studies, cohort studies, case-control studies, case series, or case reports with a minimum of five patients
3. Preclinical and clinical studies reporting clinical, functional, or metabolic outcomes of Ketogenic therapy
4. Publications available in English

**Exclusion Criteria**

1. Studies conducted exclusively in vitro or in cell-based models
2. Studies not involving a traumatic brain injury population
3. Studies without a ketogenic-based intervention
4. Duplicate reports, review articles, editorials, letters, commentaries, and conference abstracts without accompanying full data
5. Studies reporting only non-neurological or non-TBI-related outcomes
6. Publications not available in English
