# Supplementary material for: Administration of ketogenic intervention as a potential treatment during post-traumatic brain injury recovery: a scoping review
Source: Front Nutr. 2026 May 29;13:1848682. doi: 10.3389/fnut.2026.1848682 (PMC13261819; doi:10.3389/fnut.2026.1848682)
Supplement: Supplementary file 3 [file Table_3.docx]

| **Author and Year** | **Title** | **Reason for Exclusion** |
| --- | --- | --- |
| Smith et al. 2024 (18) | High-fat Diet Consumption Negatively Influences Closed-Head Traumatic Brain Injury in a Pediatric Rodent Model | No Ketogenic Intervention |
| Zhou et al. 2025 (19) | Deciphering Metabolic Responses in Traumatic Brain Injury Patients with Different Severity Using 1H NMR-Based Serum Metabolomics | No Ketogenic Intervention |
| Sullivan et al. 2004 (20) | The Ketogenic Diet Increases Mitochondrial Uncoupling Protein Levels and Activity | No TBI |
| Song et al. 2019 (21) | Integrated Proteomics and Metabolomic Analyses of Plasma Injury Biomarkers in a Serious Brain Trauma Model in Rats | No Ketogenic Intervention |
| Annoni et al. 2024 (22) | Infusion of Sodium DL-3-ß-Hydroxybutyrate Decreases Cerebral Injury Biomarkers after Resuscitation in Experimental Cardiac Arrest | No TBI |
| Seira et al. 2024 (23) | Ketone Esters Partially and Selectively Rescue Mitochondrial Bioenergetics After Acute Cervical Spinal Cord Injury in Rats: A Time-Course | No TBI |
| Morris et al. 2023 (24) | Pilot Study to Evaluate the Feasibility of Ketone Supplementation to Improve Functional Outcomes in Adolescents Post Sports-Related Concussion | Missing Manuscript |
| Appelberg et al. 2009 (25) | The Effects of a Ketogenic Diet on Behavioral Outcome after Controlled Cortical Impact Injury in the Juvenile and Adult Rat. | Duplicate |

**Supplementary Appendix C**
