# Supplementary material for: Administration of ketogenic intervention as a potential treatment during post-traumatic brain injury recovery: a scoping review
Source: Front Nutr. 2026 May 29;13:1848682. doi: 10.3389/fnut.2026.1848682 (PMC13261819; doi:10.3389/fnut.2026.1848682)
Supplement: Supplementary file 4 [file Table_4.docx]

**Supplementary Appendix D**

PRISMA flow diagram illustrating the study selection process for a scoping review examining the use of the ketogenic diet in the management of TBI. Records were identified through systematic searches of electronic databases. Following the removal of duplicates, titles and abstracts were screened for eligibility. Studies were included if they addressed ketogenic diet interventions or ketosis-related nutritional strategies in populations with TBI. The final review comprised 32 studies meeting all eligibility criteria.

**Identification of studies via databases and registers**

Records removed before screening:

Duplicate records removed

(n = 116)

Records identified from:

Databases (n = 2):

PubMed (n = 183)

Scopus (n = 238)

**Identification**

Records excluded as titles and abstracts, not relevant

(n = 269)

Records screened

(n = 305)

**Screening**

Full-text papers excluded (n = 8)

- Studies highlighting diets or mechanisms not related to ketogenic therapies (n = 4)
- Studies highlighting non-TBI injuries (n = 3)
- Duplicate study (n = 1)

Full-text papers assessed (n = 36)

Studies fulfilled the eligibility criteria (n = 28) Studies added manually (n = 3). Studies retrieved by updated search (n = 1)

**Included**

Studies included in review (n = 32)

*From:*  Page MJ, McKenzie JE, Bossuyt PM, Boutron I, Hoffmann TC, Mulrow CD, et al. The PRISMA 2020 statement: an updated guideline for reporting systematic reviews. BMJ 2021;372:n71. doi: 10.1136/bmj.n71
